# Supplementary material for: Association between oral microbiome and seven types of cancers in East Asian population: a two-sample Mendelian randomization analysis
Source: Front Mol Biosci. 2023 Nov 21;10:1327893. doi: 10.3389/fmolb.2023.1327893 (PMC10702768; doi:10.3389/fmolb.2023.1327893)
Supplement: Supplementary file 1 [file DataSheet1.ZIP › Supplementary Materials/MR plots for tongue/tongue═╝/Lung cancer/pheno.1614_to_lung cancer_forest.pdf]

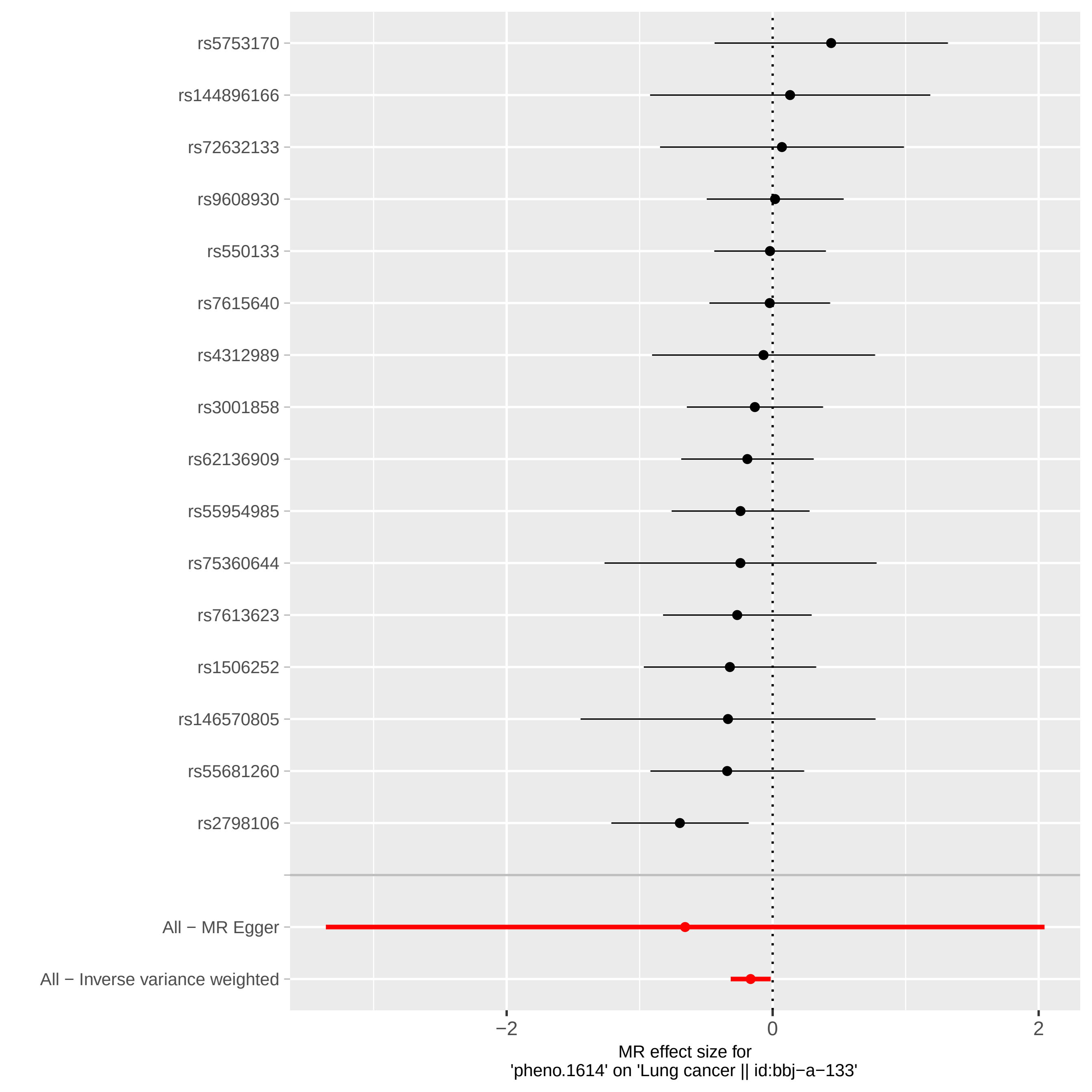

rs5753170
rs144896166
rs72632133
rs9608930
rs550133
rs7615640
rs4312989
rs3001858
rs62136909
rs55954985
rs75360644
rs7613623
rs1506252
rs146570805
rs55681260
rs2798106
All − MR Egger
All − Inverse variance weighted
−2
0
2
MR effect size for
'pheno.1614' on 'Lung cancer || id:bbj−a−133'
